# The Contribution of PGPR in Salt Stress Tolerance in Crops: Unravelling the Molecular Mechanisms of Cross-Talk between Plant and Bacteria

**DOI:** 10.3390/plants12112197

**Published:** 2023-06-01

**Authors:** Gianluigi Giannelli, Silvia Potestio, Giovanna Visioli

**Affiliations:** Department of Chemistry, Life Sciences and Environmental Sustainability, University of Parma, 43124 Parma, Italy; silvia.potestio@unipr.it

**Keywords:** plant-growth-promoting rhizobacteria (PGPR), salt stress, plant salt-responsive genes, PGPR plant gene modulation, -OMICs, holobiont–crop breeding strategies

## Abstract

Soil salinity is a major abiotic stress in global agricultural productivity with an estimated 50% of arable land predicted to become salinized by 2050. Since most domesticated crops are glycophytes, they cannot be cultivated on salt soils. The use of beneficial microorganisms inhabiting the rhizosphere (PGPR) is a promising tool to alleviate salt stress in various crops and represents a strategy to increase agricultural productivity in salt soils. Increasing evidence underlines that PGPR affect plant physiological, biochemical, and molecular responses to salt stress. The mechanisms behind these phenomena include osmotic adjustment, modulation of the plant antioxidant system, ion homeostasis, modulation of the phytohormonal balance, increase in nutrient uptake, and the formation of biofilms. This review focuses on the recent literature regarding the molecular mechanisms that PGPR use to improve plant growth under salinity. In addition, very recent -OMICs approaches were reported, dissecting the role of PGPR in modulating plant genomes and epigenomes, opening up the possibility of combining the high genetic variations of plants with the action of PGPR for the selection of useful plant traits to cope with salt stress conditions.

## 1. Introduction

The 21st century can be considered the most complex to face for agriculture, presenting a series of challenges. People must (i) produce more food to feed a growing population, with a reduction in the labor force; (ii) adopt more sustainable and efficient production methods and adapt to climate change; (iii) produce more feedstocks for a potentially huge bioenergy market; and (iv) sustain the development of the many agriculture-dependent developing countries [1]. Since the Green Revolution, the global agricultural scenario has changed drastically due to the excessive use of synthetic compounds to improve crop yields, resulting in a progressive degradation of the biological and physiochemical health of arable land and, in recent decades, a decline in agricultural productivity worldwide [2,3]. In addition, climate change will be negatively deterministic on the future state of natural resources, affecting food availability and food supply stability [4]. The main goal of the world’s agricultural system will be to increase agricultural production but under increasingly adverse conditions. Therefore, current and future dimensions require a re-thinking of some methods to decrease the impacts of agriculture on the environment and the development of sustainable technologies [5]. Soil salinity is a major abiotic stress in agricultural crop productivity worldwide and according to the Global Map of Salt Affected Soils (GSASmap) [6], more than 1.257 million hectares of soil are affected by salt and recent studies estimated that 20% of cultivated land and 33% of irrigated land is subjected to high salinity, with an expected increment of 10% per year [7,8]. The loss caused by salt soils is estimated by the agricultural sector to be USD 27.3 billion annually [9]. Crops growing in salinity-affected soils exhibit a range of morphological, physiological, and biochemical reactions, resulting in a low crop yield and quality [10].

In this context, beneficial microorganisms colonizing plant roots represent a promising tool to alleviate salt stress in various crops, thus representing a strategy for increasing agricultural productivity in saline soils [11].

In this paper, we reviewed the recent literature about (i) the roles of rhizosphere beneficial microorganisms, in particular plant-growth-promoting rhizobacteria (PGPR) in alleviating plants’ salt stress; (ii) the molecular mechanisms involved in the cross-talk between plants and PGPR; and (iii) recent -OMICs approaches to understanding molecular PGPR–plant interactions, which will open future perspectives for the application of PGPR in the field and for the microbiome-based selection of salt-tolerant crop genotypes.

## 2. Rhizosphere Microbial Community

Since their earliest evolution, land plants have been associated with a complex microbial community that includes beneficial microorganisms. The presence of beneficial microorganisms helped early land plants to respond to the environment and face challenges such as access to nutrients, new and/or stressful conditions, and pathogens [12,13].

In soil, there is a gradient of intimacy between plant roots and microorganisms, and the plant’s influence on the microbial community is greater near the root surface. A volume of soil specially influenced by plant roots or associated with the roots and plant-produced material is defined as a rhizosphere [14], a term originally coined by Hiltner [15] to describe the microorganisms of soil around and inside roots. Microorganisms living on the surface of roots are now said to inhabit the rhizosphere and the rhizoplane, and those living inside the roots are called endophytes [16,17].

Among the microbial communities colonizing the different plant regions (i.e., flowers, fruits, stems, leaves, and roots), the one associated with the roots (rhizobiome) is undoubtedly the most populated and most sophisticated of all microbial communities associated with high plants [12]. The rhizosphere effect is caused by the enormous influence exerted by the plant root exudates, a phenomenon called rhizodeposition, including a range of organic acids, amino acids, sugars, nucleosides, vitamins, and other small molecules that act as strong chemo-attractants of the soil microbiota [18]. Rhizodeposition also refers to the release into the rhizosphere of a group of specialized cells, called root cap boundary cells. This cell type is considered crucial for its effect on the rhizosphere as it usually survives after it has decayed from the roots [19,20] In particular, the passive flux of low-molecular-weight metabolites such as sugars, amino acids, and organic acids through root exudation is mostly located at the root tip, where the lack of cell differentiation favors the diffusion of metabolites to the soil [21].

The root tip senses changes in the concentration of exuded metabolites and translates them into signals to modify root growth. Through root exudate flux, plants can locally enhance concentrations of many common metabolites, which can serve as sensors and integrators of the plant nutritional status and of the nutrient availability for the rhizosphere microbial community [22,23]. Plant-associated microorganisms also constitute a strong sink for plant carbon, thereby increasing concentration gradients of metabolites and affecting root exudation. Indeed, it was found that when microorganisms were present in plant growth solution, exudation was enhanced compared to axenic conditions [24]. Rhizodeposits account for approximately 11% of the net carbon fixed by photosynthesis, and 10–16% of the total nitrogen in plants, but these values vary greatly depending on plant species and age [25]. The net sequestration of organic carbon and nitrogen by roots stimulates the multiplication of soil microbes near root tissues because (i) most known soil bacteria are organotrophic (i.e., they obtain growth energy from organic substrates) and (ii) in most soils, the access and availability of organic compounds are limited.

The microbial population colonizing the rhizosphere includes bacteria, which are the most abundant, as well as fungi, protozoa, and algae, and its composition is markedly different from that of its surrounding soil [26,27]. Regarding bacteria, Weller and Thomashow [28] proved that their presence in the rhizosphere is generally 10 to 100 times greater than in bulk soil. All plant–microbe interactions can have mutualistic or detrimental effects on the host during the microbes’ attempts to obtain nutrients and environmental protection, regardless of the physical interaction [29]. For this reason, plants need a selection mechanism that favors rhizosphere colonization by beneficial and non-pathogenic microorganisms.

The literature available suggests a two-step selection process that gradually distinguishes the root microbiota from the surrounding soil biomes and determines differences in the dimension and composition of the microbial community. In the first step, it is suggested that rhizodeposition and the recognition of the host cell wall promote organotrophic bacteria growth, thereby initiating the first community shift of the soil biome. In the second step, the microbial subpopulation undergoes a second selection process near and within the roots, based on host genotype-dependent selection, defining the profiles of the microbial communities that prosper in the rhizoplane and within the roots [19,30]. Similarly, the extent of the differences between the rhizosphere community and the soil biome may vary depending on the soil type. In addition, the specific combination of the bacterial community with which a plant reaches its best growth performance depends on the plant’s genotype and the type of soil in which it grows. Finally, microorganisms present mechanisms that allow them to recognize plant molecules, obtain nutrients, occupy space, and directly or indirectly inhibit other microorganisms in order to survive and colonize the rhizosphere [31].

## 3. Plant-Growth-Promoting Rhizobacteria (PGPR)

In 1978, Kloepper and Schroth [32] introduced the term “rhizobacteria” to indicate the soil bacterial community capable of competitively colonizing plant roots and stimulating growth, thereby reducing the incidence of plant disease. They were then renamed plant-growth-promoting rhizobacteria in 1981 [33]. PGPR represent about 2–5% of the total rhizosphere bacteria [34]. According to Nakkeeran et al. [35], an ideal PGPR should possess high rhizosphere competence, improve plant growth capabilities, have a wide range of action, be safe in relation to the environment, compatible with other rhizobacteria, and tolerant to heat, UV radiation, and oxidants.

Based on the interaction with plants and their degree of association, PGPR can be classified as either intracellular PGPR (iPGPR or symbiotic bacteria), which live inside the plants and exchange metabolites with them directly, or free-living rhizobacteria or extracellular PGPR (ePGPR), which live outside plant cells [16,36]. iPGPR include a wide range of soil bacterial genera that are usually located inside the specialized nodular structures of root cells such as *Allorhizobium*, *Azorhizobium*, *Bradyrhizobium*, *Mesorhizobium*, and *Rhizobium* of the family *Rhizobiaceae* [37]. ePGPR, instead, are present in the rhizosphere, in the rhizoplane, or in the spaces between the root cortex cells, and include genera such as *Agrobacterium*, *Arthrobacter*, *Azotobacter*, *Azospirillum*, *Bacillus*, *Burkholderia*, *Caulobacter*, *Chromobacterium*, *Erwinia*, *Flavobacterium*, *Micrococcous*, *Pseudomonas*, and *Serratia* [16]. The beneficial action of PGPR is exerted through a series of processes that result in three macro effects on the host plant: (i) increased nutrient assimilation; (ii) increased resistance to phytopathogen infections (viruses, fungi, bacteria, nematodes, insects, etc.), also referred to as biotic stresses; and (iii) increased tolerance to abiotic stresses (heavy metal in soils, drought, nutrient deficiency, salinity, temperature). This leads to improved plant performance and, consequently increased growth. The diverse mechanisms underlying these phenomena include (i) N_2_ supply through biological nitrogen fixation; (ii) the production of siderophores; (iii) the solubilization and mineralization of nutrients; (iv) the synthesis of ACC (1-aminocyclopropane-1-carboxylate) deaminase; (v) the production and modulation of phytohormones (e.g., indole 3-acetic acid (IAA), cytokinins (CK), abscisic acid (ABA), gibberellic acid (GA)); (vi) the control of plant pathogens through various mechanisms such as the secretion of enzymes able to hydrolase the fungal cell wall or compete for nutrients, the induction of systemic resistance (ISR), and the production of siderophores and antibiotics; (vii) the bio-remediation of heavy-metal-contaminated soils; and viii) an improvement in abiotic stress resistance [38,39,40,41,42].

## 4. Salt Stress Affects Plant Growth

### 4.1. Soil Salinization

Soil salinity is a major abiotic stress in agricultural crop productivity worldwide. The causes of soil salinization can be grouped into primary, due to natural sources, and secondary, which are largely anthropogenic and exacerbate the problem of salinization [9,43].

A primary source is saline parent material that releases soil mineral constituents during the chemical erosion of rock or sediment minerals, which react with air and water to produce soluble salts that are then transported away from their source of origin through watercourses. Another factor determining the development of saline soils is salty groundwater that rises through the soil profile via capillarity and releases salts, which then remain in the soil due to water evaporation. Coastal areas are particularly prone to salinization, since winds and sea spray along the coast transport salts from the sea inland in sufficient quantities to cause salinization of these areas. Sea spray can have an impact up to 80 km inland or even beyond [9]. Coastal regions are also at risk of progressive salinization due to storms, cyclones, floods, etc. Climate is another factor in the formation of saline soils, especially in arid and semi-arid regions, where evaporation results in the release of salt crystals on the soil surface. Furthermore, low precipitation affects salt leaching [43].

The main secondary cause is irrigation. Irrigation can cause salinity when salty water is used or when it causes the inadequate leaching of salts into the soil. Irrigation water can also recharge underground wells and cause them to rise, gradually introducing the salts contained in groundwater into the soil. The use of fertilized water can also contribute to the introduction of salts, which accumulate over time and increase with repeated application. Waste and wastewater are also secondary contributors to soil salinity, and so is variation in land use or land cover. In fact, alteration of the vegetation type can change the water use and evapo-transpiration characteristics of plants, with consequences such as soil drying and salt accumulation. Soil salinization is exacerbated by the excessive use of chemical fertilizers, improper irrigation practices, and industrial pollution [43]. Global warming aggravates the accumulation of salts in soils due to expanded drylands, water scarcity, and rising sea levels.

Soil is generally considered saline when the electrical conductivity (EC) of the saturation extract in the root zone exceeds 40 mM at 25 °C, with 15% of unbound Na^+^ ions [11]. Among the different ions that can cause salinization, sodium is the most soluble and widespread, and it is considered the most deleterious. In fact, unlike other ions, such as Ca^2+^, Mg^2+^, or K^+^, which plants can exploit for normal physiological and biochemical processes, Na^+^ is useless and extremely toxic to glycophytes [44].

Soils provide ecosystem services that are essential to human life and biodiversity. The salinization of soils has a serious impact on these services, as it leads to a number of consequences including (i) a reduction in agricultural productivity, water quality, soil biodiversity, and an increase in soil erosion; (ii) a decreased ability to act against contaminants as buffers and filters; (iii) a degraded soil structure; (iv) decreased functions of ecological systems such as the hydrological and nutrient cycles; (v) an increased concentration of ions that are toxic to plants; (vi) a reduced ability of crops to take up water; and (vii) reduced soil fertility and availability of micronutrients [6].

### 4.2. Plant Response to Salt Stress

Salinity stress involves changes in various plant physiological and metabolic processes, depending on the severity and duration of the stress, and ultimately inhibits plant growth. High salinity affects plants in several ways: water stress, ion toxicity, nutritional disorders, oxidative stress, the alteration of metabolic processes, membrane disorganization, a reduction in cell division and expansion, and genotoxicity [45].

It is not surprising that plants have evolved mechanisms to regulate NaCl accumulation, since NaCl is the most soluble and widespread salt. Analyses of the different growth responses under salinity conditions reveal that plants differ considerably in their tolerance [46]. Barley is the most tolerant crop among cereals, while rice is the most sensitive. Similarly, soft wheat (*Triticum aestivum*) is moderately tolerant, whereas durum wheat (*Triticum turgidum* ssp. *Durum*) is more sensitive to salt stress. Tall grass (*Thinopyrum ponticum*, syn. *Agropyron elongatum*) is a halophytic relative of wheat and is one of the most tolerant plants among monocotyledons. Indeed, among dicotyledons, the variation in salinity tolerance is even greater. For example, some legumes are very sensitive, while saltbush (*Atriplex* spp.) can grow at higher salinity concentrations than those in the sea. When compared with other species under similar conditions, *Arabidopsis* appears to be salt-sensitive, and continuous exposure to 100 mM NaCl prevents it from completing its life cycle [46,47].

An elevated salt concentration reduces shoot growth and affects plants in two main ways: it reduces the roots’ osmotic potential, thereby complicating water uptake (osmotic effect), and inside plants, it results in ion toxicity (ionic effect) [48]. A two-phase model has been proposed to describe the effects of salt stress. The osmotic phase begins immediately after the exposure of the roots to a threshold level of salinity, which is usually 40 mM NaCl or less for plants such as *Arabidopsis*. During this phase, the immediate response is stomatal closure, which also helps to reduce ion flux to the shoot; the rate of shoot growth declines, and new leaves emerge more slowly [45,46]. However, this is an unsustainable solution and is bound to be discontinued in a short time because of the difference in water potential between the atmosphere and the leaf cells, but especially due to the need to fix carbon. An interesting but as yet mechanistically unexplained phenomenon is the fact that shoot growth is more affected than root growth. A reduction in leaf growth compared to root growth would result in reduced water use, thereby maintaining soil moisture and limiting the increase in salt concentration. The second phase is determined by ion toxicity. At this stage, the response to salinity begins when salt concentration reaches toxic levels in older leaves. Unlike what happens in younger leaves, older leaves no longer expand, thus no longer dilute the incoming salt, leading to death. If the leaves die at a faster rate than they are produced, the plant’s photosynthetic capacity is compromised, and it will no longer be able to supply the carbohydrates needed by the young leaves, thereby further reducing their growth rate [46].

Usually, osmotic stress has an immediate and greater effect on growth rate than ionic stress, except for cases with a high salinity concentration or in sensitive species that do not have a strong ability to control Na^+^ transport. For most plant species, Na^+^ toxicity is greater than Cl^−^ toxicity; for this reason, almost all studies have focused on the mechanisms involved in Na^+^ control rather than Cl^−^ [46].

The main physiological, molecular, and biochemical mechanisms for survival under high salt concentration include (i) ion homeostasis and compartmentalization, (ii) ion transport and uptake, (iii) the biosynthesis of osmoprotectants and compatible solutes, (iv) the activation of the antioxidant enzyme and synthesis of antioxidant compounds, and (v) the modulation of hormones [49,50].

At the cellular level, salt tolerance is characterized by highly regulated complex mechanisms which include stress perception, and the transduction of stress signals in a cascade of molecular events leading to the activation of genes, modulating a biochemical and physiological response to obtain a substantial increase in salt tolerance [51,52].

### 4.3. PGPR Growth and Activity in Salt Stress Conditions

Most of the studies aiming to dissect PGPR activities under salt conditions are conducted in vitro, yet these observations are not directly correlated with soil in nature. Increasing NaCl concentration from 0 to 1.2 M NaCl progressively reduces the amount of ACC deaminase activity, IAA, P-solubilization, and EPS in *Bacillus pumilus* JPV11 [53]. A 38% reduction in IAA production was observed in *B. megaterium* when subjected to 5% salinity, while it increases in *P. fluorescens* cultures. When growing in 2% NaCl, *V. paradoxus* reduces the production of siderophores. No changes were observed in siderophore production for the other two strains, while a slight reduction was observed when increasing the salt concentration. There was no significant change in the ACC-deaminase activity of *P. fluorescens* and *B. megaterium* with salt concentrations, but there was a reduction in *V. paradoxus* [54].

Kusale et al. [55] conducted analyses on plant-beneficial metabolites produced by *Klebisiella variicola* under salinity stress. Increasing salt concentration determined an increase in the amount of PGP traits, although when reaching a 100 mM threshold level, phytase, siderophore, and antioxidant enzyme production was negatively impacted. A higher threshold level was observed for IAA, EPS, and ACC-deaminase activity.

Khan et al. [56] evaluated PGP traits and the survival of *Enterobacter*, *Pseudomonas*, *Rhizobium*, and *Ensifer fredii* under NaCl ranging from 1 to 5%. *Enterobacter* sp. ECD1 was the most tolerant bacterium, showing up to 50% growth in culture with 5% NaCl, followed by *Pseudomonas* spp., while *Rhizobium* spp. and *E. fredii* were the least tolerant. Compared to the other strains examined, rhizobia were the most salt-sensitive. According to the other studies, increasing levels of salinity determine a reduction in the production of IAA and siderophores. Similar results on the higher tolerance to salt stress of rhizosphere bacteria compared to endophytes were obtained when analyzing 214 isolates. Compared to endophytes, rhizosphere bacteria were more resistant, possibly due to their better adaptation to high salinity present on root surfaces [57]. A recent finding demonstrates a positive role of the two-component system (TCS) MtrAB in *Dietzia* sp. strain DQ12-45-1b in response to different environmental stresses such as hyperosmotic stress and high salinity. MtrAB is essential for the maintenance of regular cell morphology, regulating peptidoglycan metabolism and cell division under alkaline conditions [58].

Overall, increasing salt concentrations affect PGP traits and growth, but with a magnitude that seems to be related to the bacterial species and the type of PGP traits analyzed. In this regard, the most interesting PGPR to exploit in the context of plants growing in salty soil conditions are those that show unaltered growth and PGPR characteristics in response to the presence of salt.

### 4.4. Contribution of PGPR to Plant Salt Tolerance

**Figure 1:**
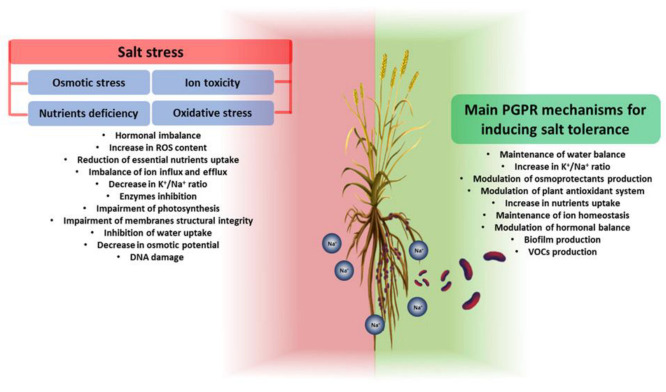
General overview of salt stress effects on plants (on the **left**) and PGPR mechanisms involved in plant salt-stress tolerance (on the **right**). Salt stress negatively affects all plant growth and development processes. Through different mechanisms, PGPR can increase plant tolerance to salt stress.

PGPR exhibit beneficial traits that allow them to mitigate the toxic effects caused by high salt concentrations. PGPR can improve plant growth in a salt environment in two main ways: (i) activating or modulating the response systems of plants during exposure to salt; and (ii) synthesizing anti-stress molecules [59]. Mechanisms to improve the growth and resistance of plants exposed to salinity include (i) the improvement of nutrient uptake (e.g., N_2_ fixation, release of bound P and K^+^ from the soil, chelating iron) and maintenance of the water balance; (ii) an influence on ion homeostasis; (iii) the induction of the selective absorption of K^+^ and exclusion of Na^+^ to maintain a high K^+^/Na^+^ ratio; (iv) the formation of biofilm to reduce Na^+^ toxicity; (v) changes in root architecture; (vi) the modulation of the antioxidant system; (vii) the modulation of osmotic substances; (viii) the modulation of plant hormonal levels; and (ix) the modulation of the expression of salt-responsive genes [60]. PGPR are classified according to their mechanisms, but in the context of salt stress the analysis of their influence on the plant’s response has shown that their promoting activity is never due to a single mechanism. In the following paragraphs, we analyze the roles of PGPR in enhancing plant salt tolerance by modulating different genes and plant cellular functions (Figure 1, and Table 1).

#### 4.4.1. Osmotic Adjustment

A decrease in osmotic pressure induces water loss and represents one of the problems plants face when growing in saline soils, because changes in osmotic pressure must be compensated for to maintain cell volume and turgor [93]. In order to maintain osmotic balance both inside and outside the cell, plants increase the synthesis of low-molecular-weight compatible solutes in the cytosol, also referred to as osmolytes. The most common osmolytes include proline, glycine betaine, soluble sugars, and ectoine [94,95]. Osmolytes are not charged, polar, and soluble, and do not interfere with cell metabolism even at high concentrations [49]. Osmotic adjustment is an energy-intensive process; in fact, it results in a reduction in growth to direct energy to osmolyte synthesis. On the other hand, it is a necessary measure to alleviate the effects of salt stress [96,97]. Proline also acts as a scavenger for stress-induced ROS; buffers the cell redox potential; and stabilizes proteins, enzymes, membrane structures, and the electron transport system complex II [98,99,100]. Moreover, proline is considered a sensitive physiological marker of salt stress.

In plants, ABA signaling and MAP kinases are involved in the production and accumulation of osmolytes [101]. The rapid activation of MAPKs such as MAPK3, 4, 6 was frequently observed in response to salt stress [102]. For instance, the salt-induced activation of MKK4 regulates the activity of MPK3, which increases the expression of stress-responsive genes such as *NCED3* and *RD29A* [103]. The protein kinase SnRK2 family can be activated in both an ABA-dependent and -independent way. SnRK2.2, SnRK2.3, and SnRK2.6 are important for transducing the ABA signal, activating AREB/ABF TFs (ABA-responsive element binding factors), and regulating the downstream responsive gene [104,105,106].

Many works show that PGPR inoculation significantly modulates the content of osmoprotectants in different plant species under salt stress. *Zea mays* inoculated with various PGPR strains was found to increase the content of osmoprotectants. As an example, the inoculation of *Enterobacter cloacae* PM23 enhances plant growth and biomass upon salt stress, increasing proline, glycine betaine, free amino acids, and soluble sugars [61].

A similar effect was found with the inoculum of *Serratia liquefaciens* KM4 under 80 and 100 mM NaCl [62]. The moderately salt tolerant *Streptomyces albidoflavus* OsiLf-2 was found to produce plenty of osmolytes, including ectoine, proline, and polysaccharides. Its inoculation in *Oryza sativa* reduces plant stress by increasing the plant’s osmotic adjustment ability [63].

The alleviation of salt stress symptoms in *Solanum lycopersicum* matched with an increase in proline content after the inoculation of *Enterobacter* 64S1 and *Pseudomonas* 42P4 [64]. *Triticum aestivum* treated with *Bacillus* sp. Wp-6 showed a rise in the content of proline, soluble sugars, and soluble proteins [65], while the inoculation of *Bacillus firmus* SWS in *Glycine max* was found to increase the proline concentration [66].

PGPR can also differentially modulate the content of different osmolytes. The content of total soluble sugars was found to have risen in *Avena sativa* plants inoculated with *Klebsiella* sp. IG 3 and grown under 100 mM NaCl, while the content of proline was reduced [67]. The same results were observed in *Zea mays* [68] and *Arabidopsis thaliana* [69] inoculated with *Bacillus* spp. and with the endophytic bacterium *Bacillus megaterium* ZS-3, respectively.

#### 4.4.2. Antioxidant Activity

Many adverse environmental factors can alter the equilibrium between ROS production and scavenging activity [107]. The content of reactive oxygen species (ROS) increases dramatically during salt stress, mainly due to the disruption of electron transport chains (ETC) during photoinhibition and/or a decrease in water potential [108]. ROS comprise different compounds such as O_2_•^−^, H_2_O_2_, ^1^O_2_, HO_2_•^−^, OH•, ROOH, ROO•, and RO•, which react spontaneously with organic molecules and cause membrane lipid peroxidation, protein oxidation, enzyme inhibition, and DNA and RNA damage [109]. In order to detoxify ROS, plants have developed antioxidant systems including antioxidant enzymes and non-enzymatic compounds. Antioxidant enzymes, such as superoxide dismutase (SOD), catalase (CAT), glutathione peroxidase (GPX), ascorbate peroxidase (APX), and glutathione reductase (GR), and the accumulation of non-enzymatic antioxidant compounds (carotenoids, flavonoids and other phenolics, proline) are positively correlated with a tolerance to salinity in plants [49].

MAPK cascades act as important signaling pathways in responding to oxidative stress and regulating ROS homeostasis. For example, the activity of scavenging enzymes is controlled by the MEKK1-MKK1/MKK2-MPK4 cascade to maintain ROS homeostasis. In *Arabidopsis*, two other proteins, MPK3 and MPK6, phosphorylate and activate HEAT SHOCK FACTOR A4A (HSFA4A), controlling ROS homeostasis and positively regulating the salt stress response [110]. Increasing evidence suggests an active role for PGPR in modulating the antioxidant defense system of plants by increasing the activity of various antioxidant enzymes under stress conditions [111,112,113]. In other studies, plants inoculated with PGPR under stress conditions showed reduced levels of enzymatic antioxidants, thereby suggesting that these plants were subject to less stress compared to uninoculated plants [114,115,116]. The application of *Acinetobacter johnsonii* SUA-14 in *Zea mays* determines a significant decline in superoxide dismutase and catalase activity, as well as in the content of MDA, an effect that might be associated with the reduction in the uptake of Na^+^ [70]. Elevated levels of antioxidants followed by a reduction in oxidative stress markers were observed in *Zea mays* inoculated with the halotolerant bacterium *Enterobacter cloacae* PM23. Under salinity stress, in inoculated plants, there was an increase in SOD, POD, APX, and ascorbic acid activity; an increase in flavonoid and phenolic content; and a reduction in H_2_O_2_ and MDA content. The rise in SOD and APX activity was also associated with an up-regulation of the SOD and APX genes [61]. Inoculation of *Pseudomonas oryzihabitans* AXSa06 in *Solanum lycopersicum* exerted a positive effect on plants grown under 200 mM NaCl, and a complex priming state was suggested by Mellidou et al. [71] to explain the difference in salt adaptation of inoculated plants. Indeed, prior to stress treatment, *P. oryzihabitans* AXSao6-inoculated plants showed increased ascorbate and MDA content, as well as an enhanced activity of POD and CAT enzymes. *Glycine max* inoculated with *Bacillus firmus* SW5 showed a lower H_2_O_2_ and MDA content under saline and non-saline conditions. Moreover, inoculated plants showed higher antioxidant activities for both DPPH and β carotene-linoleic acid assays. *B. firmus* SW5 induces the elimination of ROS by activating different antioxidant enzymes under salt stress. Furthermore, genes encoding antioxidant enzymes such as APX, CAT, Fe-SOD, and POD were up-regulated [72].

#### 4.4.3. Ion Homeostasis

Regardless of their nature, neither glycophytes nor halophytes can tolerate high concentrations of Na^+^ in their cytoplasm. The maintenance of ion homeostasis through Na^+^ ion compartmentalization is critical for growth during salt stress [45]. The regulation of Na^+^ uptake and transport in salt-stressed plants has been interpreted in the context of maintaining high K^+^/Na^+^ ratios in the cytoplasm. Because Na^+^ and K^+^ possess very similar physical and chemical properties, the two ions compete for many key metabolic processes in the cytoplasm. In fact, Na^+^ inhibits the enzymatic activity of many enzymes that require K^+^ to function. Since more than 50 different cytoplasmic enzymes are activated by K^+^, the disruption of their activity leads to a dramatic effect on cellular metabolism [117]. Na^+^ control mechanisms involve Na^+^ exclusion from roots, Na^+^ long-distance transport, and Na^+^ compartmentation [118]. Three main proton pumps have been identified as being related to salt stress tolerance in plants: (i) vacuolar-proton phosphatase which generates a proton gradient by using energy from pyrophosphate (Ppi); (ii) plasma membrane H^+^/ATPase; and (iii) vacuolar H^+^/ATPase which couples H^+^ transport and ATP hydrolysis [119]. The salt overly sensitive (SOS) stress-signaling pathway is considered the first mechanism used by plants to exclude Na^+^, and increasing evidence demonstrates its role in ion homeostasis and salt tolerance. The SOS signaling pathway is composed of three proteins: SOS3 (Ca^2+^-binding protein), SOS2 (serine/threonine kinase), and SOS1 (Na^+^/H^+^ antiporter). High salinity triggers an increase in cytosolic Ca^2+^ concentration, inducing the Ca^2+^-mediated activation of SOS3. The interaction between SOS2 and the SOS3 protein results in the activation of kinase and the release of the SOS3/SOS2 complex into the cytosol, where it phosphorylates SOS1, activating its transport function [48,120]. SOS1 is required to extract excess Na^+^ from cells (i.e., into the rhizosphere through root epidermal cells, or into the xylem through parenchyma cells of the xylem), and thus reduce ionic stress [121,122]. In addition to extruding Na^+^ from the cell through the action of SOS1, plants possess an additional system of controlling Na^+^ concentrations in the cytosol, represented by Na^+^ translocation within the vacuole. This function is performed by Na^+^/H^+^ exchangers based on tonoplasts in the NHX family. The NHX family is composed of many isoforms, differing mainly in their localization. Among all isoforms, the role of NHX1 in determining salt stress tolerance is the best characterized. As is the case with SOS1, the up-regulation of NHX1 results in greater tolerance to salt stress [123,124]. The most characterized member of HKT class I is AtHKT1 from *Arabidopsis*, the only member of the HKT family in *Arabidopsis*. AtHKT1 is involved in Na^+^ entry, and two other complementary functions have been proposed. In the phloem recirculation model, AtHKT1 loads Na^+^ into the phloem cells of the shoot and transfers it to the roots through the downward stream, thus preventing Na^+^ accumulation in the shoot. Another function of AtHKT1 is to unload Na^+^ from the xylem transpiration stream, thereby limiting the amount of Na^+^ which reached the photosynthetic tissues and supporting salt stress tolerance [117].

The *Bacillus megaterium* ZS-3 endophytic strain induces systemic tolerance to salt stress in *Arabidopsis thaliana* through the regulation of different processes, including photosynthesis, osmotic adjustment, and ion homeostasis. *B. megaterium* ZS-3 increases the K^+/^Na^+^ ratio by directly limiting the accumulation of Na^+^, rather than by increasing the K^+^ content, in both the presence and absence of salt. Furthermore, *B. megaterium* ZS-3 determines the down-regulation of *HKT1* and the up-regulation of *NHX1* and *AVP1* (a vacuolar H^+^-pyrophosphatase) pumping H^+^ into vesicles, which acidify to generate a H^+^ gradient across the membrane. These observations allow the authors to suggest a restriction of Na^+^ uptake and a sequestration of Na^+^ in plant vesicles, which enable plants to better respond to salt stress [69]. The ability of PGPR to regulate the expression of genes related to ion homeostasis was also shown by Rabhi et al. [72]. In this study, *SOS1* was found to be up-regulated in *A. thaliana* plants inoculated with *Pseudomonas knackmussii* MLR6 grown under salt conditions.

The inoculation of *Zea mays* with two strains, *Bacillus atrophaeus* WZYH01 and *Planococcus soli* WZYH02, induces better responses under salt stress, significantly increasing K^+^ and decreasing Na^+^ content, leading to a rise in the K^+^/Na^+^ ratio. Under salt stress, inoculated plants showed an up-regulation of *ZmNHX* and *ZmHKT* genes, indicating that bacterial strains might induce salt tolerance by reducing the content of Na^+^, excreting it from the cells and isolating Na^+^ in the vacuoles [73].

#### 4.4.4. Hormonal Modulation

Plants have developed several strategies to cope with salt stress by optimizing the balance between growth and stress responses, and plenty of evidence suggests a critical role for phytohormones. Abscisic acid (ABA), salicylic acid (SA), and jasmonic acid (JA) are considered stress hormones, while auxins (IAA), cytokinins (CKs), and gibberellin (GB) are considered growth promoters [125]. Most studies on hormone modulation induced by PGPR are about ABA and its induced pathways. Indeed, among all phytohormones ABA is the main factor in the regulation of resistance to abiotic stresses in plants, which coordinates a variety of functions. Its activity depends on its concentration in the plant. At a normal level, ABA regulates various physiological processes such as stomatal opening, embryo morphogenesis, seed development, dormancy, and the synthesis of storage proteins and lipids [126,127], while at high concentrations, ABA inhibits plant growth. Under conditions of abiotic stress, such as drought and salt stresses, ABA biosynthesis is strongly induced, leading to an increase in its content in the plant, and determining stomatal closure and a change in gene expression, which may favor plant adaptation and survival [128]. ABA regulates a multitude of salt-responsive genes. The comparison of *Arabidopsis* and rice transcriptomes exposed to ABA and various abiotic stresses showed that transcriptional changes affect 5–10% of the genome, and more than half were common to salinity, drought, and ABA treatments [129,130].

The importance of ABA in this phenomenon is represented by the fact that in *Arabidopsis* seedlings, the genes modulated by ABA comprise about 10% of the genome, evenly divided between induced and repressed genes, i.e., two to six times more than those modulated by other plant hormones [131]. Most ABA-modulated genes encode proteins involved in stress tolerance, such as dehydrins and enzymes that detoxify ROS, as well as those involved in osmolyte metabolism, several transporters, transcription factors, protein kinases and phosphatases, and enzymes involved in phospholipid signaling [132]. Furthermore, ABA biosynthesis is also regulated by its end products since ABA negatively regulates its own accumulation by activating its catabolic enzymes [133].

During salt stress, many transcription factors (TFs) such as MYC, MYB, bZIP, MADS, and BHLB play a fundamental role in the response to stress. TFs generally act as key negative or positive regulators of gene expression. For instance, the regulation of MYB TFs in response to salt stress involves ABA. When plants grow under high salinity conditions, the ABA content increases significantly, initiating a cascade of salt stress response signals in the ABA-dependent pathway that leads to the up- or down-regulation of downstream response genes. The specific effects are to alleviate osmotic stress and ionic stress caused by excessive salt, to maintain the water balance, and maintain the integrity of the cell membrane structure [134,135].

Ethylene is a key gaseous phytohormone with a wide range of biological activities that can affect plant growth and development. At high concentrations, it induces defoliation and other cellular processes that may lead to an inhibition of root and stem growth and premature senescence, reducing crop performance [136,137]. Under normal conditions, ethylene is produced, starting from 1-aminocyclopropane-1-carboxylate (ACC) through the action of the ACC synthetase enzyme. As a response to exposure to various environmental stresses such as drought, salt stress, cold, infections, heavy metals, and flooding, plants increase the production of ACC, resulting in a rise in ethylene concentration [31]. Auxins, such as IAA, control a wide range of processes in plant development and growth [138]. By increasing both the surface area and the length of the roots, IAA allows plants to have greater access to soil nutrients [139]. Gibberellins promote the processes of seed germination and emergence, floral induction, the development of the flower and fruit, root elongation, and lateral root extension, even if the most dominant physiological effect of GB is shoot elongation [140]. Cytokinins are involved in cell division, vascular cambium sensitivity and vascular differentiation, inducing the proliferation of root hairs, and inhibiting lateral root formation and primary root elongation [141].

PGPR can modulate plant hormones to alleviate salt stress symptoms. Most PGPR can produce IAA, thus stimulating the development of secondary roots. Furthermore, by increasing the absorption of nutrients and water, IAA alleviates the effects of some abiotic stresses such as salinity and drought [142]. The production of the ACC-deaminase enzyme is another important physiological trait of PGPR that facilitates plant growth [143,144]. Indeed, under stressful conditions, when the level of ethylene in the plant might reach inhibitory levels, this enzyme supports plant growth by degrading ACC [38,145,146].

Three different ACC-deaminase-producing *Bacillus* strains, *B. safenis* NBRI 12M, *B. subtilis* NBRI 28B, and *B. subtilis* NBRI 33N, were found to be able to modulate ethylene accumulation in *Zea mays* under salt stress conditions, reducing its content and boosting plant growth. Specifically, the application of the *B. safenis* NBRI 12M strain resulted in the maximum reduction in ACC-oxidase (ACO) activity, consistent with low ethylene production under salt stress conditions [68]. The effect of PGPR on the content of certain hormones was also found after the inoculation of *Bacillus atrophaeus* WZYH01 and *Planococcus soli* WZYH02 in *Zea mays*. The application of the two bacterial strains resulted in a reduction in ABA content and induced changes in the expression of genes related to plant hormones. In inoculated plants subjected to salt stress, gene expression analyses revealed a down-regulation of the *ZmNCED* gene and an up-regulation of the *ZmDREB2A* and *ZmWRKY58* genes, two transcription factors that play a critical role in improving plant salt tolerance [73]. Barnawal et al. [74] obtained similar results in *Triticum aestivum* inoculated with *Arthrobacter protophormiae* SA3 and *Dietzia natronolimnaea* STR1.

The inoculation of the *A. protophormiae* SA3 strain, which produces ACC-deaminase, reduces the accumulation of ABA under salt stress, a phenomenon that may be due to the reduction in the ethylene level in such plants. The damage caused by salt stress was reduced in *Citrus macrophylla* inoculated with *Pseudomonas putida* KT2440 or *Novosphingobium* sp. HR1a. The positive effects appear to be related to the rhizobacterial modulation of hormonal content; indeed, a low ABA and SA content was found. In addition, the application of *Novosphingobium* sp. HR1a was also associated with the greater accumulation of IAA in *C. macrophylla* [75]. The ABA level in leaves and roots rises in wheat grown at high salinity, determining the reduction in stomatal conductance and chlorophyll loss. *Bacillus subtilis* IB-22 increased the ABA content in the roots and suppressed the accumulation of ABA in the leaves. Since ABA is known to also maintain root extension in stressed plants, the higher content of ABA in inoculated stressed wheat can contribute to good root hydraulic conductivity, leading to better hydrated leaves [76]. In addition, *Bacillus subtilis* IB-22 produced cytokinins and raised their content in plants, thereby preventing the decline in potassium concentration in stressed plants [147]. In a related work, the role of *B. subtilis* IB-22 in the regulation of ABA content was also investigated in depth, using an ABA-deficient barley mutant (Az34) and its parental cultivar. *B. subtilis* IB22 inoculation determines an increase in ABA content in salt-stressed plant roots by supplying exogenous ABA, and by up-regulating the ABA synthesis gene *HvNCED2* and down-regulating the ABA catabolic gene *HvCYP707A1*. Inoculation resulted in a partial compensation for the mutant’s intrinsic ABA deficiency [148]. Using an *OsNAM*-overexpressed *Arabidopsis*, Tiwari et al. [77] investigated the cross-talk between salinity and phytohormones induced by *Bacillus amyloliquefaciens*-SN13 PGPR. The overexpression of *OsNAM* under salt stress resulted in the modulation of ABA, GA, and IAA, and in the regulation of APETALA2/Ethylene Responsive Factor (*AP2/ERF*), Auxin Response Factor2 (*ARF2*), salt stress-responsive genes for *GST*, and early responsive gene to dehydration (*ERD4*).

Changes in plant hormonal contents were also observed in potatoes grown under saline conditions and inoculated with *Azospirillum 14rasiliense* Sp245 or *Ochrobactum cytisi* IPA7.2, where bacterial association increased the accumulation of cytokinin and auxin in the stems and leaves [78].

*Arabidopsis* plants inoculated with *Bradyrhizobium japonicum* showed a rise in jasmonate (JA), resulting in a primed state. qPCR analyses of abiotic-stress-related and JA genes from plants pre-treated with *B. japonicum* revealed differential gene expression compared to non-inoculated plants. The up-regulation of *RD20*, *CAT3*, and *MDAR2* genes was found in *B. japonicum-*inoculated roots. In addition, under salt stress, *B. japonicum* induces a reduction in the expression of *DHAR*, *MSD1*, *MYC2*, and *RD22* in shoots and *ADC2*, *ANACO55*, *DHAR*, *GTR1*, *RD20*, *RD29B*, *VSP1*, and *VSP2* in roots [79].

12-oxo-phytodienoic acid (OPDA) is known to be a precursor of JA and related molecules, but increasing evidence suggests the role of OPDA in the regulation of plant gene expression. In particular, OPDA seems to be involved in the activation of autonomous signaling pathways, which are implicated in the regulation of a set of salt-stress-responsive genes that are not activated by JA [149,150]. In the *Prosopis strombulifera* halophyte, the OPDA content in both roots and leaves has been found to be one of the highest among all the different members of the JA family, while in the roots, the content of other JAs was reduced at high salinity, and the content of OPDA was higher and more affected by salt stress [151]. A comparative analysis of wt rice and two rice mutants impaired in ALLENE OXIDE CYCLASE (AOC) function revealed that salt stress strongly induced OPDA in wt rice and that the *aoc* mutants were less sensitive to salt stress. In mutants, the lack of 12-OPDA was correlated with lower signs of damage and with an increase in ROS scavenging activity, indicated by a rise in GTS activity and a lower MDA content [152].

Three endophytic bacteria identified as belonging to *Achromobacter* sp. (SF2) and *Bacillus* sp. (SF3, SF4), and isolated in *Helianthus annuus* L., were characterized by Forchetti et al. [153] for their ability to produce OPDA. Under normal conditions, SF2, 3, and 4 produced a very low amount of OPDA (0.01 pmol/mL), but under drought stress, the production grew significantly (11 pmol/mL). As suggested by the authors, this could help to reduce stress in plants growing under drought or salinity conditions. Erice et al. [154] provided evidence for the possibility that PGPR modulate OPDA levels, showing that in *A. thaliana*, the application of *B. megaterium* resulted in a reduction in OPDA under normal conditions, although no differences were then observed under saline conditions.

In addition, two TF genes, *WRKY50* and *ZAT10*, were modulated in the ISR induced by *Bradyrhizobium* sp. ORS278 in *A. thaliana*. *WRKY50* and *ZAT10* were previously indicated by Taki et al. [150] as OPDA-specific response genes, and were also found to be related to plant salt stress response [155,156].

An increase in OPDA content was observed only under salt stress in *Digitaria eriantha* subjected to different abiotic stress. Furthermore, plants inoculated with *R. irregularis* and subjected to salt stress showed a higher level of this metabolite than the non-inoculated ones. The higher content of OPDA in inoculated stressed plants is not correlated with the level of JA, which provides additional evidence of the role of OPDA in the regulation of genes related to the salt stress response [157]. The work cited here is not specifically about PGPR, and is rather about an arbuscular mycorrhizal symbiosis, but it offers evidence that microorganisms are capable of influencing plant growth under salt stress conditions by also modulating OPDA levels. For this reason, it would be necessary to extend the studies on plant–PGPR interaction by examining this aspect, which at present appears to be significantly neglected.

#### 4.4.5. Nutrient Uptake

A high Na^+^ concentration induces nutritional disorders that reduce the activity of many essential nutrients in the soil, making them less available to plants. Salinity reduces the uptake and translocation of nitrogen (N), potassium (K^+^), phosphorous (P), calcium (Ca^2+^), and iron (Fe) [18,158,159]. Micronutrients may be less important for plants compared to N, K^+^, Ca^2+^, and P in terms of resistance to salinity [160]. The ability to fix nitrogen is widespread among prokaryotes [161]. An estimated 80% of the biological nitrogen fixation comes from symbiotic association, while the rest is provided by free-living (diazotrophs) or associative systems [162]. Non-symbiotic nitrogen-fixing rhizobacteria mainly belong to the *Azoarcus*, *Azotobacter*, *Acetobacter*, *Azospirillum*, *Burkholderia*, *Diazotrophicus*, *Enterobacter*, *Gluconacetobacter*, and *Pseudomonas* genera [136,163]. The inoculation of biological N_2_-fixing PGPR in crops promotes plant growth, reduces the need for pesticides, and restores nitrogen levels in agricultural soil under adverse environmental conditions such as salinity [164]. After nitrogen, P is the most important element in plant nutrition. It is involved in almost all major metabolic processes, such as signal transduction, respiration, photosynthesis, macromolecular biosynthesis, and energy transfer [165]. From this perspective, phosphate-solubilizing bacteria (rhizosphere-colonizing bacteria and endophytes) play an important role in the liberation of organic phosphates or in the solubilization of insoluble inorganic phosphate [166]. The third essential and limiting plant nutrient is K^+^, which plays important functions related to enzyme activation, osmotic adjustment, and turgor generation; the regulation of membrane potential; and cytoplasmatic pH homeostasis [167]. As with P, the concentration of soluble K^+^ in the soil is very low, since 90% exists in the form of insoluble rock and silicate minerals [168]. Microorganisms are generally believed to contribute to the release of K^+^ from minerals by releasing H^+^ or, as with P, by producing organic or inorganic acids and protons (acidolysis mechanism), or by chelating ions associated with potassium minerals [112].

Fe is a key component of various metabolic pathways. More than 100 metabolic enzymes require iron as a cofactor and are essential for many plant processes, including photosynthesis, electron transport, oxidative phosphorylation, and hormone production [169]. Although it is the fourth most abundant element on earth, this huge amount of iron is not bioavailable for plants since free Fe(II) is rapidly oxidized to Fe(III), which is not assimilable due to its low solubility [170]. PGPR can produce siderophores, whose main function is to convert insoluble Fe to a form accessible to microorganisms [171]. Siderophores are also known to provide plants with iron and promote their growth [172].

Zn is one of the micronutrients required at a small concentration (5–100 mg kg^−1^) and is a co-factor and a metal activator of many enzymes [173]. Zn deficiency affects membrane integrity, and the synthesis of carbohydrates, auxins, and nucleotides [174]. Several rhizosphere bacteria potentially influence the availability of Zn for plants by solubilizing its unavailable form through the production of chelating ligands, the secretion of organic acids, vitamins and phytohormones, and through oxidoreductive systems and proton extrusion [175].

Following the inoculation of *Kosaconia radicincitans* KR-17 in *Raphanus sativus* L. grown under saline conditions, Shahid et al. [80] found the content of N, P, K^+^, Ca^2+^, Mg, Zn, Fe, Cu, and Na^+^ to rise maximally compared to non-inoculated plants. In a study on *C. quinoa*, the inoculation of the *Pseudomonas* sp. M30-35 strain mitigated salt stress, maintaining P content and homeostasis [81]. The application of *Priestia endophytica* SK1 isolated from rhizospheric soil of fenugreek in *Trigonella foenum-graecum* induced nodule formation and was found to raise the content of N and P under 100 mM NaCl compared to control plants [82]. The inoculation of *Glutamicibacter* sp. and *Pseudomonas* sp. in *Suaeda fruticose* resulted in a reduction in Na^+^ and Cl^−^ in the shoots of stressed plants. The inoculation of *Pseudomonas* sp. increased the K^+^ and Ca^2+^ content and improved the activities of urease, ß-glucosidase, and dehydrogenase compared to their corresponding non-inoculated stressed plants [83]. Comparative transcriptomic analyses between wheat roots inoculated with *Arthrobacter nitroguajacolicus* or not, revealed that under salt stress, the inoculation induces the up-regulation of various genes related to metal binding and involved in iron acquisition [84]. The application of the halotolerant *Bacillus altitudinis* WR10 strain resulted in a reduction in Na^+^ and in an increase in K^+^, P, and Ca^2+^ uptake in salt-stressed wheat, effects that can be attributed to the upregulation of *H^+^-ATPase*, and to P-solubilizing and biofilm production activity [85].

#### 4.4.6. Biofilm Formation

Biofilms are extracellular matrices composed of proteins, nucleic acids, lipids, exopolysaccharides (EPS), and embedded microorganisms, which allow rhizosphere bacteria to adhere to the surface of plant roots [176]. The biofilms produced by PGPR also protect plants from stress conditions such as drought and salinity, as their components can co-ordinately function as osmoprotectors [177]. EPS is a natural mixture of high-molecular-weight polymers released by bacteria into the environment to mitigate physiological stress and plant environmental stresses such as salinity [178]. An interesting study revealed the importance of EPS in reducing plant salt stress and colonizing roots. The exopolysaccharide-deficient *Pantoea alhagi* ΔpspD strain and the WT *P. alhagi* NX-11 were tested in hydroponic experiments to determine their ability to induce rice salt resistance. NX-11 was found to improve rice salt tolerance, while ΔpspD did not. Furthermore, the EPS produced by the NX-11 promoted the colonization of the rhizosphere by directly acting on biofilm formation and indirectly enhancing biofilm formation and chemotaxis by altering rice root exudates. qPCR analyses showed that EPS induced the up-regulation of the *OsXTH25* gene for lectin production [86]. The inoculation of *Bacillus tequilensis* resulted in the mitigation of salt stress in chickpea. *B. tequilenses* was found to exhibit bacterial flocculation directly related to EPS production and biofilm formation traits. Furthermore, Fourier-transformed infrared spectroscopy showed the presence of carbohydrates and proteins that bond to sodium ions (Na^+^) and provide salinity tolerance [87]. Exopolysaccharides also have antioxidant activity. Xiong et al. [88] investigated the production and antioxidant activity of *Glutamicibacter halophytocola* KLBMP 518 EPS and purified two EPSs (5180EPS-1 and 5180EPS-2) that showed a moderate antioxidant activity in vitro against superoxide anion and hydroxyl radical.

#### 4.4.7. Volatile Organic Compounds (VOCs)

Volatile organic compounds (VOCs) are usually a mixture of metabolites produced by microorganisms during primary or secondary metabolism, characterized by their low molecular weight (>300 Da), lipophilic nature, and low boiling point. VOCs can travel from the point of production through soils, liquid, and atmosphere, which makes them ideal chemical messengers for intra- and inter-organismic communication [179,180]. The production of VOCs by microorganisms depends on the microbial species and can be affected by many environmental factors such as pH, temperature, humidity, etc. [181].

PGPR synthesize a broad range of VOCs, including aliphatic aldehydes, esters, alcohols, organic acids, ethers, ketones, sulfur-containing compounds, and hydrocarbons. Mainly known to be involved in biocontrol activity against plant phytopathogens or the stimulation of plant defense mechanisms, VOCs produced by PGPR also affect plant growth and abiotic stress tolerance [182].

Recent works have exposed the wide range of activities exerted by VOCs in reducing salt stress in plants. Li et al. [89] analyzed the activities of VOCs produced by *Rahnella aquatilis* JZ-GX1 in *Robinia pseudoacacia* subjected to salt stress. Exposure to JX-GX1 VOCs induces changes in chlorophyll content and root morphology, an increase in the proline content and the antioxidant activity, with a correlated MDA, O_2_•^−^, and a reduction in H_2_O_2_ content. Furthermore, exposure to VOCs influences Na^+^ transport by reducing its intracellular accumulation and increasing the expression of *RpNHX1* by 99.2%.

In another work, the role of VOCs emitted by *Bacillus amyloliquefaciens* FZB42 was assessed in *Arabidopsis* grown under 100 mM NaCl. Plants grown under salt conditions and treated with FZB42 VOCs showed an increase in both chlorophyll content and total soluble sugars. The activity of antioxidant enzymes such as CAT, SOD, and POD was found to have risen, determining an augmented ROS scavenging capacity. Moreover, the expression of *HKT1* and *NHX1* was induced by FZB42 VOCs. Interestingly, the utilization of *Arabidopsis jar1-1* and *myc2* mutant lines suggested that VOCs produced by FZB42 might alleviate salt tolerance by regulating JA signaling pathways [90]. A reduction in oxidative stress markers such as ROS, MDA and proline was also found in *A. thaliana* grown under 100 mM NaCl exposed to VOCs produced by *Burkholderia pyrrocinia* CNUC9. Dimethyl disulfide (DMDS), methyl thioacetate, and 2-undecanone were identified as products of CNUC9 and their role in alleviating salt stress was confirmed using respective synthetic compounds [91].

*Paraburkholderia phytofirmans* PsJN was found to increase plant tolerance to diverse abiotic stresses, including salinity. Interestingly, the early exposure of *A. thaliana* to *P. phytofirmans* VOCs for 11 days induces long-term effects, resulting in salinity tolerance, even when the plants were transplanted into a standard culture throughout the entire plant life cycle. An analysis of organic compounds revealed the production of 2-undecanone, 7-hexanol, 3-methylbutanol, and dimethyl disulfide, and, as in the previous cases, the application of a blend composed of their corresponding synthetic compounds was found to mimic the effects of PsJN on salinity tolerance [92].

## 5. Future Perspective for Dissecting Plant–Microbe Molecular Interactions under Salt Stress

The possibility of exploiting PGPR in the open field to alleviate the symptoms of salt stress in plants requires more in-depth studies for the development of bio-inoculants that are effective in agricultural soils with high amounts of salt.

For this purpose, it is necessary to characterize the growth strategies under stress conditions of microbiota from different salt-stressed environments, and the molecular mechanisms commonly employed in attributing to plants the ability to tolerate harsh edaphic conditions. Broad-spectrum research is also needed to identify salt-resistant PGPR in order to produce bio-inoculant formulations that can be functional in the open field. Additionally, -OMICs techniques can be useful to identify metabolites and genes involved in the mitigation of salt stress, not only in plants but also in bacteria that are able to tolerate extreme saline environments. To this end, plant-associated microbiota can influence multiple plant regulatory cascades which together define the plant’s phenotype, but as yet, little is known about the global modulation of gene expression in plants under salt stress conditions by PGPR, and about the signal molecules involved in cross-talk between plants and bacteria.

The possibility of performing comparative analyses using -OMICs techniques (e.g., metagenomics, transcriptomics, proteomics, and metabolomics) in plant tissues and in microbes during interaction with their hosts could allow a deeper understanding of the mechanisms at the basis of the salt tolerance response induced by PGPR.

**Figure 2:**
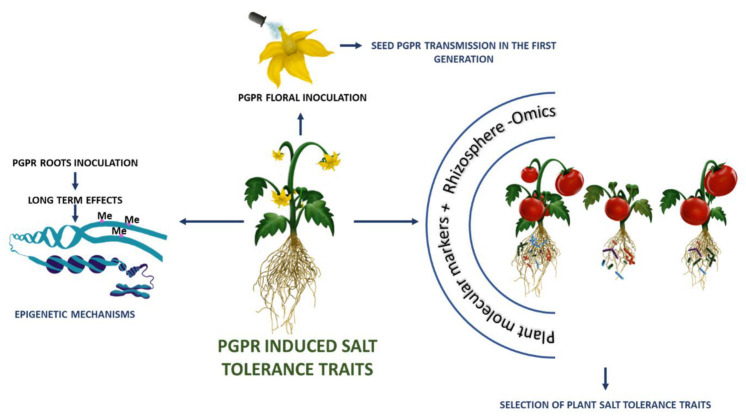
Holobiont-level breeding strategies for the selection of salt tolerant genotypes. Association between individual changes in DNA methylation (novel epialleles) and changes in phenotype induced by PGPR; PGPR inoculation in plant reproductive organs modify phenotypic traits of the progeny, thus acquiring the desired phenotype obtained through a combination of host- and microbial-encoded traits; use of metagenomics information to map the host genetic determinants of the rhizosphere microbiota for the selection of plant salt tolerance traits.

Interestingly, PGPR could also alter the plant’s epigenome, reprogramming its global transcription patterns. As an example, a transcriptomic approach was utilized to identify the genes expressed in tomato roots at different times after inoculation with the beneficial fungus *Trichoderma harzianum* T22 [183]. The authors showed that the beneficial fungus induced transcriptome reprogramming, epigenetic modifications, and alternative splicing in the tomato root tissues [183]. The modification of the root transcription pattern due to PGPR inoculum is also associated with possible epigenetic mechanisms, which are also part of the plant’s response to biotic and abiotic stress (Figure 2) [184]. Some stress-induced epigenetic modifications may be stable and heritable, becoming epigenetic memories in the process of plant adaptation to stresses [185,186]. Very recently, it has been shown that PGPR inoculation causes the modification of DNA methylation, which regulates the expression of genes useful for the promotion of plant growth, and that these DNA methylation changes in the DNA of root tissues can persist even if the PGPR is removed from the root microbiome [187]. This research opens up the possibility for PGPR to shape the epigenome of the plants, contributing to the phenotypic plasticity caused by epigenetic variation that affects fitness and, eventually, natural selection in plants. Recent studies have also highlighted that plant interaction with PGPR is highly dependent on plant genotype, and there is a scientific debate on whether selection strategies for modern varieties have negatively impacted on the capacity of plants to interact with PGPR [188,189,190]. Interestingly, genetically identical plants, displaying distinct epigenomes, can differentially alter their microbiota, thus being a source of variability for crop improvement based on microbial communities [191].

For this purpose, the holobiont-level breeding strategy has been proposed, in which microbes are one of the direct targets of the selection process that help to achieve a desired plant phenotype, even in response to abiotic stress (Figure 2) [192]. For example, the possibility to induce epimutations by applying an exogenous demethylation agent would be a fast method to generate variability that could alter the assembly of plant rhizosphere microbiota [193].

In addition, microbiota abundance in the rhizosphere assessed by metagenomics allowed the mapping of the host genetic determinants of the rhizosphere microbiota in wild and domesticated genotypes of barley [194], thereby demonstrating that the heritable component of the plant microbiota in the rhizosphere is controlled by a relatively low number of loci, similar to other plant species [195,196].

Another interesting approach involves the introduction of PGPR into seeds to modify the plant seed microbiome. It involves introducing a microbial strain into the parent plant during flowering (Figure 2). After seed germination, the PGPR strain multiplies and spreads through the new plant tissues of the next generation. This approach was successfully performed by Mitter and collaborators [197], by introducing the endophyte *Paraburkholderia phytofirmans* PsJN into the flowers of soybean and pepper. This method drove the inclusion of the bacterial strain in the progeny seed microbiome, thus inducing vertical inheritance to the offspring generation.

## 6. Conclusions

In conclusion, in many works described in this review, PGPR have been shown to be effective in improving plant growth under salinity. Thanks to these bacteria’s ability to grow under salt stress conditions and their effects on plant growth, they are a very interesting, sustainable solution for improving crop productivity in saline soils. Although some PGPR have been associated with the modulation of genes belonging to different signal transduction pathways such as *MYC*, *DREB2*, and *WRKY*, extensive research remains to be conducted to understand the signaling pathways involved in plant and PGPR cross-talk, inducing the plant’s salt tolerance response. In addition, the possibility of applying PGPR consortia in the open field for the cultivation of crops in saline soils needs more in-depth studies, to verify the efficacy of PGPR formulations on different plant species or genotypes. Furthermore, the use of -OMICs approaches to explore the highly genetic and epigenetic variations of plants combined with the action of PGPR could be useful for the selection of plant phenotypes able to cope with environmental issues such as salt stress.

## Figures and Tables

**Table 1:** Examples of studies showing the effect of rhizosphere microbes on plant responses to salt stress.

PGPR	Plant Species	PGPR-Induced Plant Salt Stress Responses	References
Osmotic adjustment			
*Enterobacter cloacae* PM23 *Serratia liquefaciens* KM4	*Zea mays*	↑ Proline, glycine betaine, free amino acids, and soluble sugars	[61,62]
*Streptomyces albidoflavus* OsiLf-2	*Oryza sativa*	↑ Proline, total soluble sugars	[63]
*Enterobacter* 64S1 *and Pseudomonas* 42P4	*Solanum lycopersicum*	↑ Proline	[64]
*Bacillus sp.* Wp-6	*Triticum aestivum*	↑Proline, soluble sugars, and soluble proteins	[65]
*Bacillus firmus* SWS	*Glycine max*	↑ Proline	[66]
*Klebsiella* sp. IG 3	*Avena sativa*	↑ Total soluble sugars; ↓ Proline	[67]
*Bacillus* spp.	*Solanum lycopersicum*	↑ Total soluble sugars; ↓ Proline	[68]
*Bacillus megaterium* ZS-3	*Arabidopsis thaliana*	↑ Total soluble sugars; ↓ Proline	[69]
Antioxidant activity			
*Acinetobacter Johnsonii* SUA-14	*Zea mays*	↓ Superoxide dismutase and catalase activity, MDA content	[70]
*Enterobacter cloacae* PM23	*Zea mays*	↑ SOD, POD, APX, ascorbic acid activity, flavonoid and phenolic content ↓ H_2_O_2_ and MDA content	[61]
*Pseudomonas oryzihabitans* AXSa06	*Solanum lycopersicum*	*↑* Ascorbate and MDA content, POD, and CAT activity	[71]
*Bacillus firmus* SW5	*Glycine max*	*↓* H_2_O_2_ and MDA content; ↑ APX, CAT, Fe-SOD, and POD	[66]
Ion homeostasis			
*Bacillus megaterium* ZS-3	*Arabidopsis thaliana*	*↑* K^+/^Na^+^ ratio, *NHX1* and *AVP1; ↓ HKT1*	[69]
*Pseudomonas knackmussii* MLR6	*Arabidopsis thaliana*	*↑* SOS1	[72]
*Bacillus atrophaeus* ZYH01 *Planococcus soli* WZYH02	*Zea mays*	*↑ K*^+^/*Na*^+^ ratio, *NHX*, and *HKT*	[73]
Hormonal modulation			
*Bacillus safenis* NBRI 12M *Bacillus subtilis* NBRI 28B *Bacillus subtilis* NBRI 33N	*Zea mays*	*↓* Ethylene production, ACC-oxidase activity	[68]
*Bacillus atrophaeus* WZYH01 *Planococcus soli* WZYH02	*Zea mays*	*↓* ABA content, *NCED* gene; *↑ DREB2A* and *WRKY58* genes	[73]
*Arthrobacter protophormiae* SA3 *Dietzia natronolimnaea* STR1	*Triticum aestivum*	*↓* ABA content; *↑ DREB2*	[74]
*Pseudomonas putida* KT2440 *Novosphingobium* sp. HR1a	*Citrus macrophylla*	*↓* ABA, SA and IAA content	[75]
*Bacillus subtilis* IB-22	*Triticum durum*	*↓* ABA content in the roots, *HvNCED2; ↑* ABA content in the leaves, *HvCYP707A1*	[76]
*Bacillus amyloliquefaciens* SN13	*Arabidopsis*	*↑ OsNAM;* ↑/↓ ABA, GA, IAA, *AP2/ERF*, *ARF2*, *GST*, and *ERD4*.	[77]
*Azospirillum brasiliense* Sp245 *Ochrobactum cytisi* IPA7.2	*Solanum tuberosum*	*↑* Cytokinin and auxin in the stems and leaves	[78]
*Bradyrhizobium japonicum*	*Arabidopsis thaliana*	*↑ RD20*, *CAT3*, and *MDAR2* genes in roots; ↓ *DHAR*, *MSD1*, *MYC2*, and *RD22* in shoots and *ADC2*, *ANACO55*, *DHAR*, *GTR1*, *RD20*, *RD29B*, *VSP1*, and *VSP2* in roots	[79]
Nutrients uptake			
*Kosakonia radicincitans* KR-17	*Raphanus sativus* L.	*↑* N, P, K^+^, Ca^2+^, Mg, Zn, Fe, Cu, and Na^+^	[80]
*Pseudomonas* sp. M30-35	*Chenopodium quinoa*	Maintaining P content and homeostasis	[81]
*Priestia endophytica* SK1	*Trigonella foenum-graecum*	*↑* N and P	[82]
*Glutamicibacter* sp. *Pseudomonas* sp.	*Suaeda fruticose*	↓ Na^+^ and Cl^−^ in shoots	[83]
*Pseudomonas* sp.	*Suaeda fruticose*	*↑* K^+^ and Ca^2+^, activity of urease, ß-glucosidase, and dehydrogenase	[83]
*Arthrobacter nitroguajacolicus*	Wheat	*↑* Genes related to metal binding and involved in iron acquisition	[84]
*Bacillus altitudinis* WR10	Wheat	↓ Na^+^; *↑* K^+^, P, and Ca^2+^, *H^+^-ATPase*, P-solubilizing activity and biofilm production	[85]
Biofilm production			
*Pantoea alhagi* NX-11	*Zea mays*	*↑ OsXTH25*	[86]
*Bacillus tequilensis*	*Cicer arietinum*	Bacterial flocculation directly related to EPS production and biofilm formation traits	[87]
*Glutamicibacter halophytocola* KLBMP 518	*-*	Antioxidant activity in vitro against superoxide anion and hydroxyl radical	[88]
Volatile organic compounds (VOCs)			
*Rahnella aquatilis* JZ-GX1	*Robinia* *pseudoacacia*	Changes in chlorophyll content and root morphology; *↑* Proline and antioxidant activity; ↓ MDA, O^2^•^−^, and H_2_O_2_; ↓ intracellular Na^+^; *↑ RpNHX1*	[89]
*Bacillus amyloliquefaciens* FZB42	*Arabidopsis thaliana*	*↑* Chlorophyll content and total soluble sugars; *↑* CAT, SOD, POD, and ROS scavenging capacity; *↑ HKT1* and *NHX1*; alleviate salt-tolerance-regulating JA signaling pathways	[90]
*Burkholderia pyrrocinia* CNUC9	*Arabidopsis thaliana*	↓ ROS, MDA, and proline	[91]
*Paraburkholderia phytofirmans* PsJN	*Arabidopsis thaliana*	Long-term effects resulting in salinity tolerance	[92]

**↑ =** increase in content or increase in gene expression; **↓ =** decrease in content or decrease in gene expression.

## Data Availability

Data are contained within the article and in the cited references.
